# Construction of Nanofibrillar Networked Wood Aerogels Derived from Typical Softwood and Hardwood: A Comparative Study on the In Situ Formation Mechanism of Nanofibrillar Networks

**DOI:** 10.3390/molecules29050938

**Published:** 2024-02-21

**Authors:** Wenjing Yan, Yan Qing, Zhihan Li, Lei Li, Sha Luo, Ying Wu, Deng Chen, Yiqiang Wu, Cuihua Tian

**Affiliations:** College of Materials Science and Technology, Central South University of Forestry and Technology, Changsha 410004, China; yanwenjing2656@163.com (W.Y.); qingyan0429@163.com (Y.Q.); lizhlive@163.com (Z.L.); elevenair@163.com (L.L.); luosha0713@163.com (S.L.); wuying2000g@outlook.com (Y.W.); chendeng0102@163.com (D.C.)

**Keywords:** typical softwood, typical hardwood, wood aerogel, nanofibril network, formation process

## Abstract

The construction of networks within natural wood (NW) lumens to produce porous wood aerogels (WAs) with fascinating characteristics of being lightweight, flexible, and porous is significant for the high value-added utilization of wood. Nonetheless, how wood species affect the structure and properties of WAs has not been comprehensively investigated. Herein, typical softwood of fir and hardwoods of poplar and balsa are employed to fabricate WAs with abundant nanofibrillar networks using the method of lignin removal and nanofibril’s in situ regeneration. Benefiting from the avoidance of xylem ray restriction and the exposure of the cellulose framework, hardwood has a stronger tendency to form nanofibrillar networks compared to softwood. Specifically, a larger and more evenly distributed network structure is displayed in the lumens of balsa WAs (WA-3) with a low density (59 kg m^−3^), a high porosity (96%), and high compressive properties (strain = 40%; maximum stress = 0.42 MPa; height retention = 100%) because of the unique structure and properties of WA-3. Comparatively, the specific surface area (SSA) exhibits 25-, 27-, and 34-fold increments in the cases of fir WAs (WA-1), poplar WAs (WA-2), and WA-3. The formation of nanofibrillar networks depends on the low-density and thin cell walls of hardwood. This work offers a foundation for investigating the formation mechanisms of nanonetworks and for expanding the potential applications of WAs.

## 1. Introduction

Aerogel, a super-lightweight and porous material, is obtained by replacing the liquid component in a gel with gas, and the framework structure remains intact without considerable collapse [1]. In 1931, Kistler [2] published the first article on aerogels, which reported ideas on constructing aerogels and the first successful preparation of a silica aerogel using the sol–gel method and supercritical drying. In the early 21st century, a new generation of aerogel materials, called bio-aerogels [3], emerged and garnered immense attention. These materials were primarily derived from biomass, such as polysaccharides. Compared to brittle silica aerogels, bio-aerogels exhibit higher flexibility, higher fracture resistance, lower density, and higher surface area. Bio-aerogels combine the advantageous qualities of inorganic aerogels and synthetic polymer aerogels, rendering them highly versatile in applications such as thermal insulation, sensors, energy storage, and pollutant treatment [4,5,6,7,8,9].

Wood aerogel (WA) is a type of bio-aerogel material derived from wood and is appreciated for its outstanding qualities, such as having a lightweight, flexible, and porous structure. These unique characteristics have resulted in its widespread application in various fields, including insulation, adsorption, acoustics, energy storage, environmental remediation, and biomedicine [8,10,11,12,13,14,15]. This material, combining the properties of inorganic and organic aerogels, is eco-friendly because of its renewability, degradability, and biocompatibility. Thus, it plays a crucial role in the efficient use of forestry resources and the development of advanced materials through value-added applications. Recently, there has been a rapid development of WAs, which has demonstrated a promising potential across various fields. Regulating wood components resulted in intriguing structural and physicochemical changes. After delignification, wood demonstrates a notably high specific surface area (SSA) and microporous/mesoporous structures. It can be modified easily with abundant -OH groups, which is attractive for functional modification. Furthermore, the delignified wood (DW) can be used as an aerogel in multiple industries. DW was employed as a matrix by Wang et al. [16], and it was modified with polyethyleneimine to create wood-based aerogel materials with a high selective adsorption capacity. The physicochemical properties were improved by further leveraging the natural structure of the wood channels and creating a network structure within them, thereby expanding their potential applications in biomedical, environmental protection, nanocomposite materials, nanoelectronic devices, and other fields. In 2011, Li [17] reported the use of ionic liquids for the top-down disassembly of all-component wood and the preparation of regenerated lignocellulosic aerogels using the freeze–thaw approach. Garemark et al. [11] used an ionic liquid mixture to prepare strong shape-memory bio-aerogels from natural balsa, birch, ash, and spruce. He et al. [18] used nanotechnology and carboxylated functionalization treatment approaches to create an eco-friendly, tunable, dual-wavelength absorption mesoporous balsa WA. These results paved the way for research on aerogels derived from agroforestry biomass. The use of 2,2,6,6-tetramethylpiperidinyl-1-oxide (TEMPO)-mediated oxidation in balsa wood had been previously investigated in numerous studies [19,20]. The mesoporous structure and SSA were improved by the dense cell wall, which contained nanofibrillar networks [21,22]. The development of a new product derived from nanowood was reported by Yang et al. [23]. To achieve mechanical/thermomechanical multistability, balsa wood was treated through TEMPO-mediated oxidation. In conclusion, the exclusivity of balsa wood was discovered by previous researchers. However, what are the unique advantages of balsa wood, and in what ways do other wood species have limitations in the preparation of WAs in terms of structure and performance? This is a pressing question that needs to be addressed. Therefore, this work considers the impact of wood species, density, and cell wall thickness on the formation of WAs.

Herein, the impact of wood species, density, and cell wall thickness on WA formation was investigated using a two-step top-down method. The SSA of fir WAs (WA-1), poplar WAs (WA-2), and balsa WAs (WA-3) was increased 25-fold, 27-fold, and 34-fold, respectively. In addition, the aerogels exhibited porosities of approximately 85% for WA-1, 77% for WA-2, and 96% for WA-3. High elasticity was achieved due to the confinement of nanofibrillar networks within pores. The maximum compressive strength at ε = 40% strain was approximately 0.4 MPa for WA-1, 0.09 MPa for WA-2, and 0.1 MPa for WA-3. Furthermore, through the delamination of ultrathin layers and the fibrillation action of the TEMPO-NaClO_2_-NaClO (TNN) system on cellulose, a microscopic nanofibrillar network structure was formed in situ. Moreover, the effects of wood species, density, and cell wall thickness on the structure and performance of WAs were compared, providing a research foundation for the application of WAs in environmental protection and energy development [24,25]. The formation process of WAs at the microscopic level was investigated, providing a research foundation for a deep understanding of WAs and their formation mechanism, which plays an important role in the advanced utilization of wood.

## 2. Results and Discussion

### 2.1. Preparation of WAs

**Figure 1:**
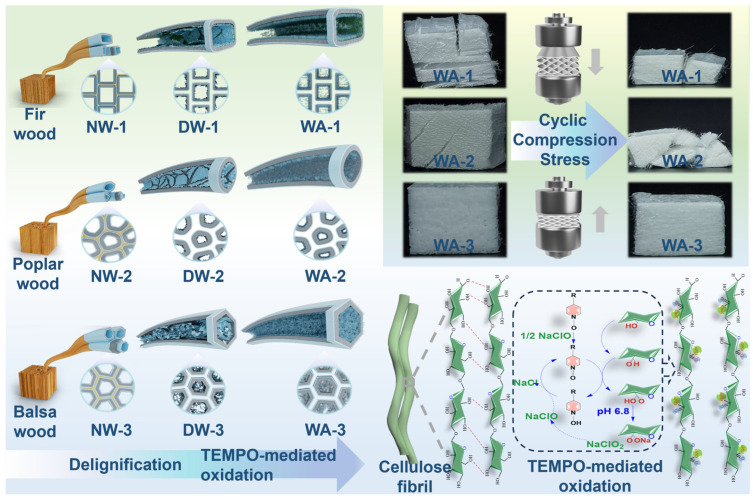
Schematics of the preparation of fir, poplar, and balsa wood aerogels (WAs). The left diagram shows the sequential steps involved in the production of WAs using wood materials sourced from three species, each with a different density and cell wall thickness. The upper-right corner visually represents the mechanical strength characteristics of the three WAs, whereas the lower-right corner provides a visual representation of the formation of nanofibrillar network structures.

To prepare WAs, natural fir (NW-1, density ~374 kg m^−3^), poplar (NW-2, density ~442 kg m^−3^), and balsa (NW-3, density ~76 kg m^−3^) wood were selected as the starting materials for their various species, density, and cell wall thickness (Figure 1). NW-1, -2, and -3 were submerged in a buffer solution of 2 wt% NaClO_2_ and C_2_H_3_NaO_2_ with a pH of 4.6 at 80 °C for 12 h to prepare DWs. Delignification occurred through the radical action of ClO_2_, which oxidized aromatic rings and caused ring-opening reactions that broke down the lignin. The natural structure of the cellulose fibers was preserved using this process, thereby maintaining the mechanical strength of the DW [12]. A TNN-mediated oxidation chemical treatment at pH 6.8 was used to form WAs. This treatment resulted in the mild disintegration of the cellulose in solution. The C6 primary hydroxyl group was selectively oxidized to form a C6 carboxylic acid group, leading to the in situ formation of cellulose nanofibers via electrostatic repulsion between anionic cellulose microfibrils [26]. Subsequently, 50 wt% tert-butanol was introduced as an antisolvent. The intermolecular hydrogen bonds were reinstated, which led to the transformation of the dissolved cellulose chains and the in situ formation of solid nanofibrillar structures within the lumens. The nanofibrillar networks were exclusively formed within the wood pores and were not allowed to diffuse into the regeneration bath for several reasons. Firstly, the cell wall contained tiny pores that restricted the formation of networks. Secondly, there was an interaction between the nanofibers and cell walls, limiting the space for network structure formation. Finally, the wood structure shrunk during solvent exchange, which prevented the diffusion of nanofibrillar networks outside the wood [13]. Even after drying, the nanofibrillar networks in the lumen space remained intact due to the high concentration of fibrils and the rapid freezing rate [27]. The lumens filled with nanofibers have a substantially large SSA, which was confirmed by analyzing the N_2_ adsorption data.

### 2.2. Morphology and Structure

**Figure 2:**
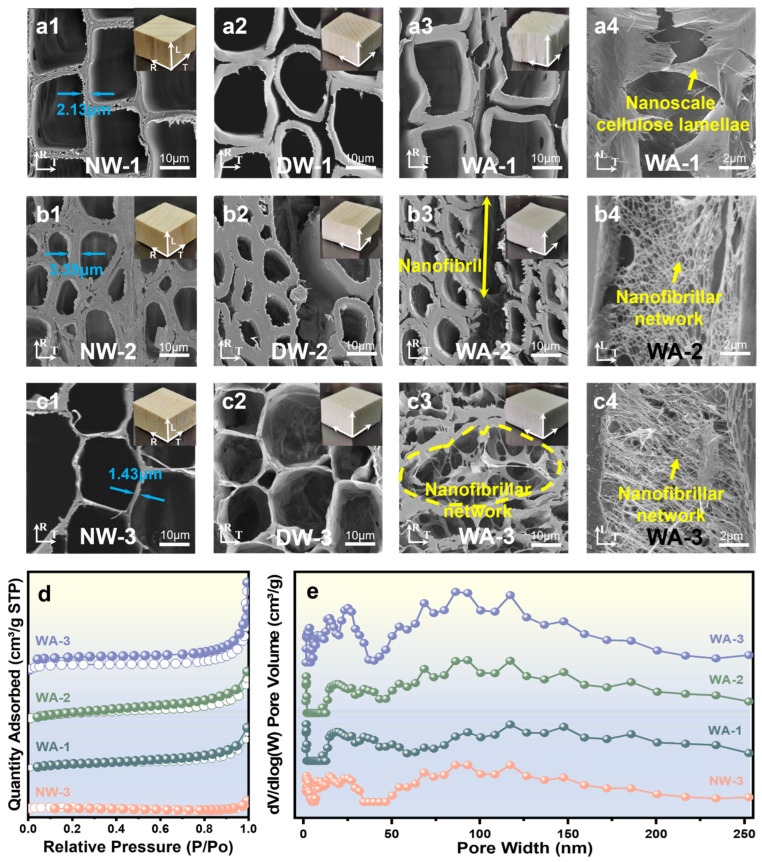
Morphology and structure of various wood samples. SEM images of the cross-sections of (**a1**) NW-1, (**a2**) DW-1, (**a3**) WA-1, (**b1**) NW-2, (**b2**) DW-2, (**b3**) WA-2, (**c1**) NW-3, (**c2**) DW-3, and (**c3**) WA-3, as well as the surfaces along the fiber direction of (**a4**) WA-1, (**b4**) WA-2, and (**c4**) WA-3. (**d**) Nitrogen physisorption isotherms and (**e**) an image of the pore size distributions of NW-3, WA-1, WA-2, and WA-3.

NW-1 was a representative softwood characterized by an organized quadrangle cellular structure with thin cell walls (~2.13 μm) (Figure 2(a1)). Meanwhile, NW-2 was categorized as a hardwood with an oval cell structure and exhibited thicker cell walls (~3.39 μm) and smaller cell pores (Figure 2(b1)). Alternatively, NW-3 exhibited a honeycomb-like hexagon structure with even thinner cell walls (~1.43 μm) and larger cell pores (Figure 2(c1)). The cell wall of wood mainly comprised cellulose microfiber bundles within the lignin and hemicellulose matrix [28]. Both the appearance and structure of DW were considerably different from those of unprocessed NW because of the partial removal of lignin and hemicellulose. This removal resulted in the delamination of cell wall layers and the formation of pores in the previously lignin-rich secondary S1 wall of DW [29]. After delignification, cells of delignified fir wood (DW-1) were completely detached from each other (Figure 2(a2)), massive fractures appeared between certain cells of delignified poplar wood (DW-2) (Figure 2(b2)), and tiny gaps formed between neighboring cells of delignified balsa wood (DW-3) (Figure 2(c2)). In addition, delignification resulted in the in situ formation of nanoscale holes in the cell wall [30]. After TNN-mediated oxidation, the resulting aerogels had a macroscopic appearance similar to that of DW but exhibited a unique nanostructure. The SEM images of the WA cross-section (Figure 2(a3–c3)) and surface along the fiber direction (Figure 2(a4–c4)) revealed that the lumen space of the fiber was occupied by nanofibrillar networks. The development of these networks within the lumen was influenced by wood species, density, and cell wall thickness. At the lumen/cell wall interface of WA-1, nanoscale cellulose lamellae in the cell wall layer and a limited number of nanofibrillar networks were observed (Figure 2(a3,a4)). WA-2 showed detached nanofibers from the cell wall layer in the lumen, leading to sparse nanofibrillar networks (Figure 2(b3,b4)). The regenerated nanofibrillar network extended across the lumen space of WA-3, with nanofibrillar structures filling all the space of micropores in the fiber cross-sections and the cell wall corners (Figure 2(c3) and Figure S1 (Supplementary Materials)). A dense and consistent nanofibrillar network structure developed on the surface along the fiber direction (Figure 2(c4)). Comparatively, the network structure created by hardwood in poplar and balsa wood was notably dense, with balsa wood being particularly superior.

Comparing typical softwood and hardwood with similar densities, fir wood was more prone to layering, highlighting the thin layer structure of cell walls in the cell lumens. This indicated that delignification resulted in a more severe degradation of the cell walls due to their thin cell walls and simple lignin chemical composition [29]. Relatively, network structures could be constructed in poplar wood lumens but lacked density and uniformity. This indicated that the chemical reagent could not fully penetrate the cell walls because of the thickness of the cell walls and the complex lignin chemical composition. It was observed that the formation of nanofibrillar networks was substantially impacted by the thickness of the cell walls and the lignin chemical composition. Comparing typical hardwood with different densities, it was evident that the cell lumens of balsa wood were filled with dense and orderly arranged nanofibrillar networks, distinguishing it from poplar. This showed that the proportion and density of the networks depended on the density of the original wood. Fir and balsa wood, which had thinner cell walls, exhibited easy peeling and dispersion of cell walls, underscoring the crucial influence of cell wall thickness on the preparation of WAs.

The SSA of WAs was investigated using the Brunauer–Emmett–Teller (BET) method. It was demonstrated that the porosity of WAs was substantially improved by reducing the matrix content and creating an intracellular network structure in situ (Figure 2d and Figure S2). Wood with higher porosity typically resulted in aerogels with larger pore volumes and surface areas. The BET surface areas of WA-1, WA-2, and WA-3 were 29.4, 37.8, and 82.3 m^2^ g^−1^, respectively. These values were approximately 25, 27, and 34 times higher than those of NW-1 (1.2 m^2^ g^−1^), NW-2 (1.4 m^2^ g^−1^), and NW-3 (2.4 m^2^ g^−1^). Furthermore, abundant mesopores were observed in WAs with sizes of 10–30 nm, while the macropore size remained virtually unchanged (Figure 2e). The porous structure of WAs played a crucial role in the preparation of functional wood materials and served as a structural foundation for the functional application of wood.

### 2.3. Chemical and Structural Analysis

**Figure 3:**
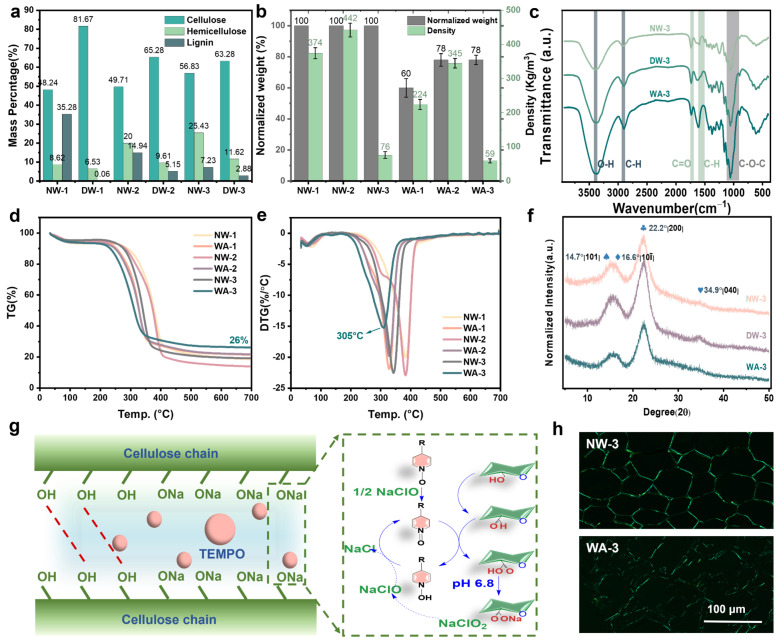
Chemical characteristics of various wood samples. (**a**) Cellulose, hemicellulose, and lignin contents of NW-1, NW-2, NW-3, DW-1, DW-2, and DW-3. (**b**) Weights and densities of NW-1, NW-2, NW-3, WA-1, WA-2, and WA-3. (**c**) Fourier transform infrared spectra of NW-3, WA-1, WA-2, and WA-3. (**d**) TG and (**e**) DTG curves of NW-1, NW-2, NW-3, WA-1, WA-2, and WA-3. (**f**) X-ray diffraction spectra of NW-3, WA-1, WA-2, and WA-3. (**g**) Schematic of the TEMPO-NaClO_2_-NaClO-mediated oxidation system acting on the cellulose chain. (**h**) Polarizing microscopic image of NW-3 and WA-3.

The contents of cellulose, hemicellulose, and lignin were measured to investigate changes in the chemical composition. The observations showed that cellulose was better preserved in WAs. However, approximately 99.8%, 66%, and 60% of lignin were successfully removed from NW-1, NW-2, and NW-3 (Figure 3a) during the delignification treatment. This removal process also eliminated the light-absorbing chromophoric compounds mainly derived from lignin, resulting in a white texture. The average material yields of WAs after delignification, selective oxidation, and FD were approximately 60, 78, and 78 wt%, respectively. In addition, the density of these materials decreased from initial values of approximately 374, 442, and 76 kg m^−2^ to approximately 224, 345, and 59 kg m^−2^ (Figure 3b). These findings indicate the efficiency of lignin removal, and fir wood displayed the highest removal rate. This is attributed to the simpler and easier-to-remove lignin structure of softwood [30]. 

The groups of NWs, DWs, and WAs were comparatively analyzed using the Fourier transform infrared spectra (FT-IR) to confirm the successful removal of lignin and partial carbohydrates (Figure 3c and Figure S3). The characteristic absorption peaks of lignin ranged from 1596 to 1505 cm^−1^ (C-H) [31] and the broadening of the C-H peak indicated modifications in certain lignin structures. Furthermore, cellulose exhibited characteristic absorption peaks at 1200–950 (C-O-C), 3400 (-OH), and 2900 (C-H) cm^−1^ [32]. The intrinsic bonds of cellulose were mostly unchanged, indicating that the cellulose molecules were not considerably affected by TNN treatment. The decrease in the C=O peak at 1732 cm^−1^ (representing acetyl groups) indicated structural changes in hemicellulose, such as deacetylation or hemicellulose removal [12].

Figure 3d,e present the thermogravimetric analysis (TG) and differential thermogravimetry (DTG) curves of NWs and WAs under an N_2_ atmosphere at 700 °C. The TG peak shifted toward higher temperatures as the heating rate increased due to the hysteresis. The active pyrolysis zone, characterized by substantial weight loss, occurred approximately between 150 °C and 600 °C. Cellulose, hemicellulose, and lignin underwent strong depolymerization within this range [33]. Above 600 °C, the passive pyrolysis zone was observed where weight loss occurred gradually, lignin degradation continued, charring occurred, and residual ash and fixed carbon were produced [34]. Table 1 shows the specific characteristic temperatures of the sample. The DTG curve demonstrates that the largest peak appeared in the temperature range of 250–400 °C, indicating that this was the main temperature range for the pyrolysis reaction [35]. The temperature range of the active pyrolysis zone was consistent for NWs and WAs. It was widely recognized that biomass materials typically had similar primary components and, thus, exhibited comparable pyrolysis mechanisms. This was believed to result from the superposition of their compositional mechanisms [36]. Comprehensive analysis revealed that WA-3 demonstrated the highest residual quality and optimal thermal stability. This indicated the superior performance of WA-3 in terms of its ability to withstand high temperatures without notable degradation.

To investigate the partial dissolution process in cellulose, powder X-ray diffraction analysis (XRD) was conducted (Figure 3f and Figure S4). All WAs underwent substantial changes in their crystal structures. The mechanism of dissolution can be inferred by examining the intensity variations of the characteristic peaks of crystalline natural cellulose I (2θ = 14.7°, 16.6°, 22.8°, and 34.9°) and amorphous cellulose I (2θ = 21.5°) [37]. As revealed using the peak deconvolution method, WAs exhibited broader amorphous peaks and lower intensities of cellulose I peak. The broader absorption bands observed indicate the absence of a well-defined order or crystallinity in the WA structure. The lower intensities of the cellulose I peak can be attributed to a lower cellulose concentration in the WAs or changes in cellulose composition, leading to reduced peak intensity. A slight shift toward lower angles in the [200] peak of the WAs was observed (Figure 3f). Although the exact reasons for this phenomenon are not completely understood, it may be due to an increase in amorphous cellulose near the crystalline cellulose. According to the results reported by Abe and Yamamoto [38], the expanded wood matrix mechanically interacts with the cell wall microfibril during the drying process and could cause peak shifts. Notably, hydroxyl groups on the cellulose chains could be selectively converted into hydroxy aldehydes or hydroxy acids after TNN-mediated oxidation (Figure 3g). Introducing these functional groups could result in changes in the physicochemical properties of cellulose, such as enhanced solubility, improved solubilization, and increased reactivity. This, in turn, provided the necessary conditions for the in situ formation of nanofibrillar networks inside the WA cell lumens. A polarizing microscope is a crucial tool for investigating the morphology of polymer crystals. More accurate and detailed information about crystallization could be obtained using a polarizing microscope. NW-3 exhibited a dense honeycomb-like crystalline structure, while WA-3 underwent deformation in its crystal structure with numerous gaps appearing between the cell walls (Figure 3h and Figure S5). This promoted flexibility and elasticity of WAs while preserving the natural alignment of the wood channels. Therefore, a high mechanical performance was retained. This illustrated the advantages of the two-step approach in regulating the physicochemical properties of WAs, as it allowed for the alteration of the crystal structure while preserving the mechanical characteristics. 

### 2.4. Mechanical Properties

**Figure 4:**
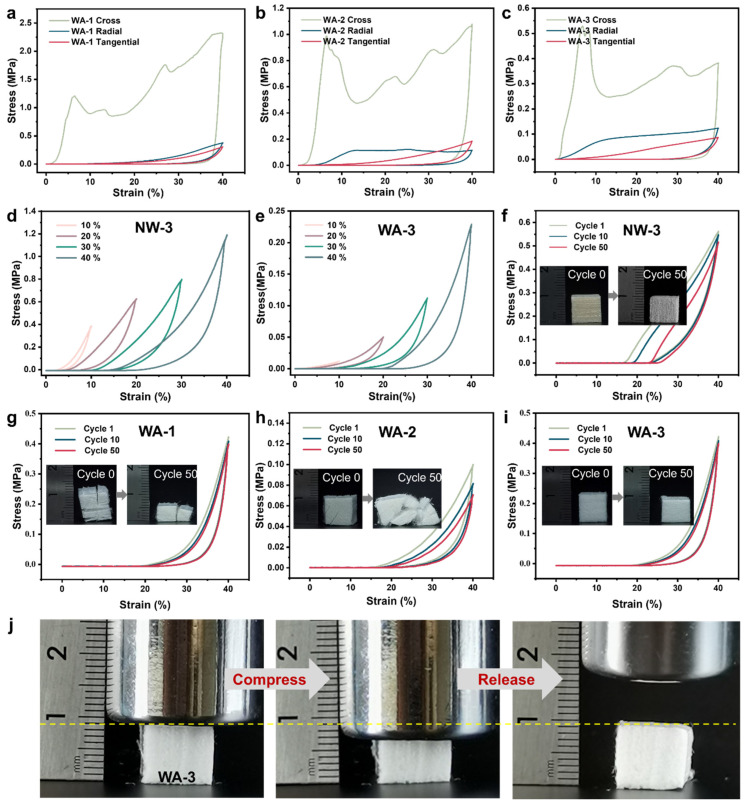
Mechanical compressibility and fatigue resistance of the NW-3 and WAs. Stress–strain curves of (**a**) WA-1, (**b**) WA-2, and (**c**) WA-3 under compression of cross, radial, and tangential sections. Stress–strain curves of (**d**) NW-3 and (**e**) WA-3 under compression at maximum strain values of 10%, 20%, 30%, and 40%. Stress–strain curves of (**f**) NW-3, (**g**) WA-1, (**h**) WA-2, and (**i**) WA-3 under cyclic compression at a maximum strain of 40%. (**j**) Photographs of WA-3 showing its reversible compressibility along the layer stacking direction.

Figure 4 and Figure S6 illustrate the compression properties of NWs and WAs, which exhibited a distinct plateau region as the strain became 20%. In this region, an increase in strain was observed, while the stress levels remained stable. The reason may be the ability of dislocations and slip mechanisms in the material to counteract the external strain, ensuring its structural stability. The presence of this plateau region clearly demonstrated significant plastic deformation. In the following region, a rapid increase in stress was observed, resulting in linear densification. This densification phenomenon occurred when voids and stacked layers were progressively eliminated under additional loading [14]. 

The preservation of the anisotropy of NW was apparent in WAs, and this characteristic was demonstrated in compressive experiments (Figure 4a–c). The compressive performance was greatest in the tangential section, while it was weakest in the cross-section for all three aerogels. Figure 4d,e illustrate the compressive stress–strain behavior of NW-3 and WA-3 materials, displaying their response to maximum compressive strains of 10%, 20%, 30%, and 40%, respectively. Several compression tests were conducted involving 50 loading–unloading cycles at a constant strain rate of 40% to assess the fatigue resistance. The results indicated that the most notable plastic deformation was experienced by NWs after undergoing 50 compression cycles (Figure 4f and Figure S7). The thin cell wall of WA-1 exhibited increased elasticity, but it was prone to fracturing under cyclic compression and released due to the presence of softwood rays (Figure 4g). However, WA-2, being a hardwood aerogel, had thicker cell walls and larger gaps due to delignification, leading to a higher plastic deformation and a reduced height retention. Eventually, WA-2 was crushed after undergoing 50 compression cycles (Figure 4h). WA-3 demonstrated excellent mechanical compressibility and fatigue resistance (Figure 4i). This can be attributed to its unique spring-like lamellar structure, which includes many large pores embedded between the curved layers. Such a specific structure enabled the substantial deformation of WA-3 without localized structural collapse [14,39]. Notably, WA-3 exhibited an impressive ability to withstand a compressive strain of up to 40% and exceptional mechanical compliance and could return to its original height after releasing the stress (Figure 4j). 

### 2.5. Formation Mechanism of the Nanofibrillar Network

**Figure 5:**
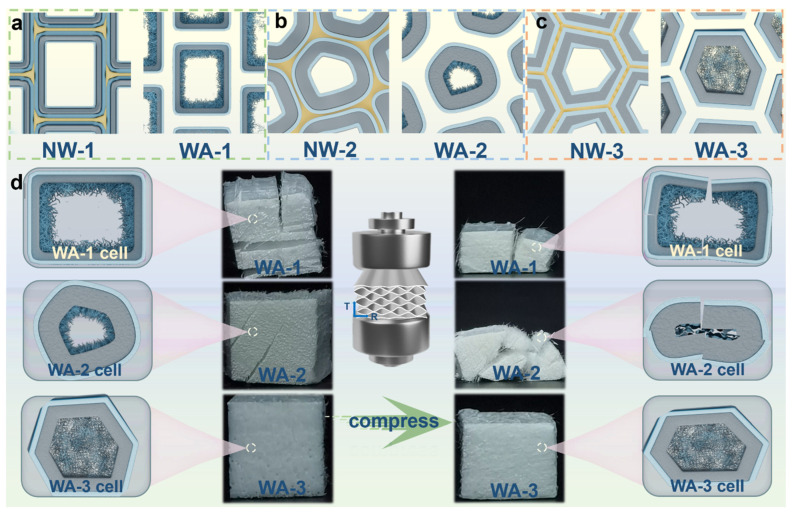
The cell wall thickness of WAs and their response to transverse compression. Schematics of (**a**) NW-1 and WA-1, (**b**) NW-2 and WA-2, (**c**) NW-3 and WA-3, (**d**) the cell walls of WA-1, WA-2, and WA-3 under transverse compression.

The structure and physicochemical properties of WAs were substantially influenced by the inherent characteristics of wood. Notably, WAs from softwood and hardwood exhibited significant differences in network structure. Softwood was particularly prone to cracking and deformation due to xylem rays, resulting in a reduced mechanical strength (Figure 5a). Conversely, hardwood overcame these drawbacks and emerged as an exceptional material choice for WAs (Figure 5b,c). In addition, a thinner wood cell wall and lower density promoted a more uniform nanofibrillar network structure in WAs, leading to enhanced physical and chemical properties (Figure 5c). However, thicker cell walls and a higher density led to a subpar structure and performance (Figure 5b) [40]. The deformation behavior of the microstructure of WAs has been speculated based on the lateral compression deformation characteristics of WA-1, WA-2, and WA-3 (Figure 5d) [23]. The cell wall framework architecture and nanofibrillar network of WA-1 and WA-2 showed clear signs of structural collapse as they were gradually compressed. WA-2, which had a dense and thick cell wall, experienced a more pronounced plastic deformation and an uneven stress distribution. In contrast, WA-3, which possessed a lightweight cell wall framework architecture and dense nanofibrillar network structure, exhibited a distinctive elastic geometric deformation and a uniform stress distribution as it was gradually compressed.

**Figure 6:**
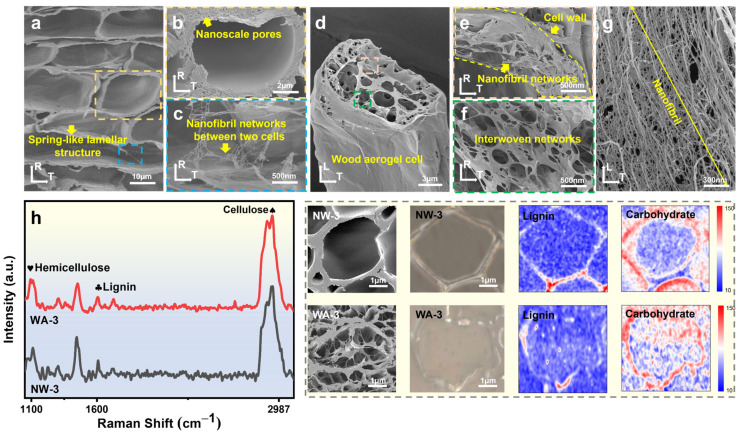
(**a**) Low- and (**b**,**c**) high-magnification SEM images of the cross-sections of WA-3. SEM images of (**d**) a single cell of WA-3, (**e**) nanofibrillar networks in WA-3 cell wall, (**f**) interwoven networks in the cell lumen, and (**g**) nanofibers of network structure. (**h**) Confocal Raman spectroscopy and images of NW-3 and WA-3.

The in situ formation mechanism of nanofibrillar network in WAs can be understood by analyzing the cellulose aggregation mechanism of wood at the molecular and supramolecular levels. In the primary and secondary cell walls, cellulose microfibrils were arranged in a layered structure within the cell wall, offering strong mechanical reinforcement. These microfibrils were intricately wound and deposited layer by layer in the wood cell wall. Furthermore, the various components of the cell wall interacted with each other through noncovalent bonds, forming a cohesive and well-defined supramolecular structure. Specifically, in the secondary S2 layer, cellulose, hemicellulose, and lignin combined to create a tightly stacked ultrathin layer [41]. It was possible to selectively remove lignin and hemicellulose while preserving most of the cellulose using a gentle sodium chlorite-acetate treatment. This treatment disrupted the hydrogen bonds between cellulose, hemicellulose, and lignin, resulting in the solubilization of the cell wall [42]. The TNN-mediated oxidation process targeted the C6 primary hydroxyl group of cellulose to convert it into carboxylic acid groups [19,20,26]. The thin and fractured cell walls tended to adhere to the nearest intact cell wall during the FD process, leading to a distinctive lamellar structure resembling a spring (Figure 6a) [23]. Numerous nanopores (Figure 6b) emerged in the cell wall under the combined effect of sodium chlorite-acetate and TNN-mediated oxidation [43]. The spring-like lamellar structure and cell walls were interconnected with nanofibrillar network structures (Figure 6c). For a single WA cell (Figure 6d), the exposed cellulose on the cell wall appeared to form an interwoven nanofibril network structure in situ under the effect of TNN-mediated oxidation fibrillation (Figure 6e). The intracellular structure comprised a woven network formed by ultrathin layers (Figure 6f), which were shaped by the directed arrangement and intertwining of nanofibrils (Figure 6g). This indicated that the TNN-mediated oxidation process not only facilitated reactions within individual cellulose molecules but also facilitated interactions between neighboring molecules [44]. Therefore, the oxidation reaction could propagate throughout the wood, establishing a vast network structure of aerogel pore channels.

Figure 6h showed a comparison of Raman images and the spectra of the cross-sections of NW-3 and WA-3. The peak at approximately 1600 cm^−1^ indicated the presence of lignin, while peaks at approximately 2897 and 1100 cm^−1^ were characteristic peaks of carbohydrates of cellulose and hemicellulose [42,45]. Compared with that of NW-3, the lignin signal peak of WA-3 was notably weakened, and the carbohydrate signal peaks had partially disappeared. This result indicated the successful removal of partial lignin and hemicellulose using a sodium chlorite-acetate solution. Raman images provided visual representations of the relative amounts of cellulose and lignin in different regions of the middle lamella, cell wall, and lumen nanonetwork structures [42]. In NW-3, cellulose, hemicellulose, and lignin were highly concentrated in the cell wall, whereas their concentrations were very low in the cell lumen. Conversely, in WA-3, a considerable decrease in lignin concentration was observed. Moreover, high concentrations of carbohydrates (mostly cellulose) were observed in the cell wall and cell lumen. This demonstrated that WA-3 successfully formed a cellulose network in the cell lumen, with small amounts of lignin and hemicellulose.

## 3. Materials and Methods

### 3.1. Materials and Chemicals

Fir wood (*Cunninghamia lanceolata* (*Lamb.*) *Hook*, 450 ± 50 kg/m^3^) was selected as the representative softwood species. Poplar wood (*Populus. simonii Carr*, 454 ± 56 kg/m^3^) and balsa wood (*Ochroma pyramidale*, 113 ± 30 kg/m^3^) were chosen as the representative hardwood species. All wood species were cut into dimensions of 10 × 10 × 10 mm^3^ (tangential × radial × axial). Sodium chlorite (80%, NaClO_2_), sodium acetate anhydrous (99%, C_2_H_3_NaO_2_), sodium hypochlorite (Cl ≥ 5.2%, NaClO), acetic acid (99%, C_2_H_4_O_2_), and tert-butanol (99%, C_4_H_10_O) were provided by Sinopharm Chemical Reagent Co., Ltd. (Shanghai, China). Additionally, 2,2,6,6-tetramethylpiperidinyl-1-oxide (98%, C_9_H_18_NO) was supplied by Shanghai Yuanye Bio-Technology Co., Ltd. (Shanghai, China). It is important to note that all the chemicals used in the experiment were of analytical grade.

### 3.2. Preparation of DW

Natural fir, poplar, and balsa wood (NW-1, NW-2, and NW-3) (10 × 10 × 10 mm^3^ (tangential × radial × axial)) were subjected to the delignification process using 2 wt% sodium chlorite-acetate in an acetate buffer (pH 4.6). The delignification process took place over a period of 12 h at a temperature of 80 °C. Following delignification, the delignified fir wood (DW-1), delignified poplar wood (DW-2), and delignified balsa wood (DW-3) were carefully rinsed with deionized water (DI water). Table 2 shows the relative content of cellulose, hemicellulose, and lignin of NW and DW.

### 3.3. Preparation of WAs

DW (1 g) was added to a 0.05 mol/L phosphate-buffered solution (90 mL, pH 6.8) in a closed reaction vessel. The TNN-mediated oxidation system was mixed according to the mass ratio of TEMPO (0.016 g, 0.1 mmol): NaClO_2_ (1.13 g, 10 mmol): NaClO (1.5 mL, 1.0 mmol). The container was sealed immediately, and the temperature was set to 60 °C for 2 h and assisted by ultrasound for 2 h. After that, a 50 wt% tert-butanol solution was added, followed by freeze-drying (FD) for 24 h. Table 3 shows the relative content of sodium carboxylate and crystallinity of NW, DW, and WAs.

### 3.4. Characterization

The morphologies of NWs, DWs, and WAs were analyzed using field emission scanning electron microscopy (SEM, ZEISS Sigma 300, Carl Zeiss Jena, Oberkochen, Germany). To improve sample conductivity, a gold coating was applied using the sputtering coater (SC7620, Quorum Technologies, East Sussex, United Kingdom) for 45 s at 10 mA. The acceleration voltage during topography shooting was set at 3 kV. The specific surface area (SSA, ASAP 2460) was then measured under liquid nitrogen conditions to conduct nitrogen absorption and desorption tests on the samples. The analysis of NWs and DWs was evaluated according to the NY/T 3492. The cellulose, hemicellulose, and lignin analysis was evaluated using the NY/T 3494-2019 standard method. The apparent volumetric mass density of the NWs and WAs was determined by weighing the samples and measuring their pores. Dimensions and weights were measured using a digital caliper (PD-151, accuracy: 0.01 mm, Pro’sKit, Taiwan, China) and electronic scales (AR124CN, accuracy: 0.0001 g, Aohaosi, Shanghai, China). Equation (1) was utilized to determine the porosity, with the solid density of wood assumed to be 1500 kg/m^3^ [46].

(1) $$porosity=1-\frac{density\;of\;sample\;[kg/m^{3}]}{solid\;density\;of\;sample\;[kg/m^{3}]}$$

Fourier transform infrared spectra were recorded using a spectrometer (FT-IR, Nicolet iN10, Thermo Fisher Scientific, Franklin, MA, USA) with a scan range of 400–4000 cm^−1^. To assess thermal properties, the samples were subjected to thermogravimetric analysis and differential thermogravimetry (TG and DTG, TG 209 F1, Netzsch, Bavaria, Germany) analyses. The temperature range for the analysis was set at 30–700 °C, with a heating rate of 20 °C/min. Powder X-ray diffraction (XRD, Empyrean, Malvern Panalytical, Almelo, Holland) scans were performed over 2θ of 5–50° with a step size of 10°. Prior to analysis, the samples were pretreated for 12 h under a vacuum of 90° using the standard degasifier of Mack Instruments. The polarizing characteristics of the materials were tested using a professional polarizing microscope (DM2700P, Leica, Wetzlar, Germany). Mechanical compressibility was evaluated using a microcomputer-controlled electronic universal testing machine (CMT6502, Sansiyongheng, Zhejiang, China).

The sample cross-sections were embedded in an Lr White Resin on a glass slide. The confocal Raman microscope (CRM, *α*300 R, WITec, Ulm, Germany) with a laser (*λ* = 532 nm, laser power = 20 mW; WITec, Ulm, Germany) was focused through a 100 × Zeiss objective onto the embedded microsections and collected the Raman scattering signal back through the same objective, passing a band-pass filter before being guided via an optic multifiber (i.d. = 50 μm) to the spectrometer (300 g mm^−1^ grating, UHTS 300 WITec, Ulm, Germany). A complete wavenumber spectrum was obtained at each pixel, and the integration time was 0.5 s.

## 4. Conclusions

This work focused on the construction and mechanism of aerogels derived from fir, poplar, and balsa wood. Highly porous and elastic aerogels were successfully formed through a two-step top-down process. Lignin and hemicellulose were removed and TNN-mediated oxidation nanofibrillar networks within the lumen micropores were formed in situ. Comparatively, the specific surface area (SSA) exhibited 25-, 27-, and 34-fold increments in cases of WA-1, WA-2, and WA-3. The enhancement in elasticity varied among the three types of WAs, with WA-3 exhibiting high porosity (up to 96%), elastic ability (ε = 40%; maximum stress = 0.42 MPa), and deformation recovery rate (100%). Hardwood exhibited a more complete structure and better mechanical properties than softwood owing to the influence of xylem rays and lignin components. Comparing the two types of hardwood, density directly influenced the penetration of chemicals into cells, whereas the cell wall thickness affected the depth and breadth of chemical action. This led to better cell lumen morphological structures in WA-3, which has a lower density and a thinner cell wall. This work offered valuable insights into the preparation approach and in situ formation mechanism of WAs and contributed to the advancement of wood-based materials.

## Figures and Tables

**Table 1:** Characteristic temperature and index of NW and WA pyrolysis.

Sample	DTG*_max_* (°C)	C*_f_* (wt%)
NW-1	383.4	−81.29
WA-1	328.9	−78.49
NW-2	382.9	−86.03
WA-2	328.9	−78.19
NW-3	342.5	−80.80
WA-3	309.9	−73.85

DTG*_max_*: Maximum mass loss rate. C*_f_*: Mass change at the final temperature.

**Table 2:** Relative content of cellulose, hemicellulose, and lignin of NW and DW.

Sample	Cellulose (%)	Hemicellulose (%)	Lignin (%)
NW-1	48.2385	8.6179	35.2846
DW-1	81.6735	6.5331	0.0562
NW-2	49.7124	20.0036	14.9353
DW-2	65.2821	9.6136	5.1515
NW-3	56.8344	25.4290	7.2296
DW-3	63.2754	11.6244	2.8822

**Table 3:** Relative content of sodium carboxylate and crystallinity of NW, DW, and WAs.

Sample	Relative Content of Sodium Carboxylate (%)	Crystallinity (%)
NW-1	0.7	80.21
DW-1	1.1	46.76
WA-1	4.7	74.58
NW-2	0.8	86.20
DW-2	1.2	58.82
WA-2	4.5	98.58
NW-3	1.2	83.33
DW-3	1.2	61.19
WA-3	5.3	89.81

## Data Availability

Data are contained within the article and supplementary materials.

## Supplementary Materials

The following supporting information can be downloaded at: https://www.mdpi.com/article/10.3390/molecules29050938/s1, Figure S1: (a) Photograph of NW-3. (b) High magnification SEM image of NW-3 cell wall. (c) High magnification SEM image of NW-3 cell corner (CC). (d) Photograph of DW-3. (e) High magnification SEM image of DW-3 cell wall. (f) High magnification SEM image of DW-3 CC. (g) Photograph of WA-3. (h) High magnification SEM image of WA-3 cell wall. (i) High magnification SEM image of WA-3 CC.; Figure S2. Porosity of NW-1, WA-1, NW-2, WA-2, NW-3, and WA-3.; Figure S3. FT-IR spectra of (a) NW-1, DW-1 and WA-1. (b) NW-2, DW-2 and WA-2. (c) NW-3, DW-3 and WA-3. (d) WA-1, WA-2 and WA-3.; Figure S4. XRD spectra of (a) NW-1, DW-1 and WA-1. (b) NW-2, DW-2 and WA-2. (c) NW-3, DW-3 and WA-3. (d) WA-1, WA-2 and WA-3.; Figure S5. Polarizing microscopic image of (a) NW-3, (b) DW-3 and (c) WA-3.; Figure S6. NW-3, DW-3 and WA-3 under cyclic compression at the maximum strain of 40%.; Figure S7. Mechanical compressibility of NWs. Photographs of the (a) NW-1 with 0 loading-unloading cycles. (b) NW-1 with 50 loading-unloading cycles. (c) NW-2 with 0 loading-unloading cycles. (d) NW-2 with 50 loading-unloading cycles. (e) NW-3 with 0 loading-unloading cycles. (f) NW-3 with 50 loading- unloading cycles. Reference [47] is cited in the Supplementary Materials.

## Abbreviations

NW	natural wood
DW	delignified wood
WA	wood aerogel
FD	freeze-drying
TNN	TEMPO-NaClO_2_-NaClO

## References

[1] Alemán J.V., Chadwick A.V., He J., Hess M., Horie K., Jones R.G., Kratochvíl P., Meisel I., Mita I., Moad G. (2007). Definitions of terms relating to the structure and processing of sols, gels, networks, and inorganic-organic hybrid materials (IUPAC Recommendations 2007). Pure Appl. Chem..

[2] Kistler S.S. (1931). Coherent Expanded Aerogels and Jellies. Nature.

[3] Budtova T. (2019). Cellulose II aerogels: A review. Cellulose.

[4] Hu P., Wang J., Zhang P., Wu F., Cheng Y., Wang J., Sun Z. (2022). Hyperelastic Kevlar Nanofiber Aerogels as Robust Thermal Switches for Smart Thermal Management. Adv. Mater..

[5] Wu Y., Zhao Y., Zhou M., Tan S., Peymanfar R., Aslibeiki B., Ji G. (2022). Ultrabroad Microwave Absorption Ability and Infrared Stealth Property of Nano-Micro CuS@rGO Lightweight Aerogels. Nano-Micro Lett..

[6] Sun X., Yu Q., Wang F., Hu S., Zhou J., Liu Y., Jiang Z., Wang X., Yu Y., Yang H. (2024). Sustainable lignocellulose aerogel for air purifier with thermal insulation, flame retardancy, mechanical strength, and its life cycle assessment. Int. J. Biol. Macromol..

[7] Dong H., Li X., Cai Z., Wei S., Fan S., Ge Y., Li X., Wu Y. (2023). Strong, Lightweight, and Shape-Memory Bamboo-Derived All-Cellulose Aerogels for Versatile Scaffolds of Sustainable Multifunctional Materials. Small.

[8] Xie T., Wang Y., Zhang Q., Shen S., Guo W., Chen X., Wang Q., Qu L., Li C. (2024). Wood aerogels decorated amino-functionalized MIL-101(Cr) as efficient filter for multistage purification of wastewater. Chemosphere.

[9] Wang Z. (2023). Preparation of Wood Gel Materials and Their Anisotropic Applications. Ph.D. Thesis.

[10] Dong X., Gan W., Shang Y., Tang J., Wang Y., Cao Z., Xie Y., Liu J., Bai L., Li J. (2022). Low-value wood for sustainable high-performance structural materials. Nat. Sustain..

[11] Garemark J., Perea-Buceta J.E., Felhofer M., Chen B., Cortes Ruiz M.F., Sapouna I., Gierlinger N., Kilpelainen I.A., Berglund L.A., Li Y. (2023). Strong, Shape-Memory Lignocellulosic Aerogel via Wood Cell Wall Nanoscale Reassembly. ACS Nano.

[12] Garemark J., Perea-Buceta J.E., Rico Del Cerro D., Hall S., Berke B., Kilpelainen I., Berglund L.A., Li Y. (2022). Nanostructurally Controllable Strong Wood Aerogel toward Efficient Thermal Insulation. ACS Appl. Mater. Interfaces.

[13] Garemark J., Yang X., Sheng X., Cheung O., Sun L., Berglund L.A., Li Y. (2020). Top-Down Approach Making Anisotropic Cellulose Aerogels as Universal Substrates for Multifunctionalization. ACS Nano.

[14] Guan H., Cheng Z., Wang X. (2018). Highly Compressible Wood Sponges with a Spring-like Lamellar Structure as Effective and Reusable Oil Absorbents. ACS Nano.

[15] Li M., Fu S. (2021). Photochromic holo-cellulose wood-based aerogel grafted azobenzene derivative by SI-ATRP. Carbohydr. Polym..

[16] Wang C., Biswas S.K., Okubayashi S. (2020). Polyethylenimine-Impregnated Mesoporous Delignified Wood with High Mechanical Strength for CO2/N2 Selective Adsorption. ACS Appl. Nano Mater..

[17] Li J., Lu Y., Yang D., Sun Q., Liu Y., Zhao H. (2011). Lignocellulose aerogel from wood-ionic liquid solution (1-allyl-3-methylimidazolium chloride) under freezing and thawing conditions. Biomacromolecules.

[18] He W., Cao J., Guo F., Guo Z., Zhou P., Wang R., Liang S., Pang Q., Wei B., Jiao Y. (2023). Nanostructured carboxylated-wood aerogel membrane for high-efficiency removal of Cu (II) ions from wastewater. Chem. Eng. J..

[19] Isogai A., Saito T., Fukuzumi H. (2011). TEMPO-oxidized cellulose nanofibers. Nanoscale.

[20] Saito T., Hirota M., Tamura N., Isogai A. (2010). Oxidation of bleached wood pulp by TEMPO/NaClO/NaClO2 system: Effect of the oxidation conditions on carboxylate content and degree of polymerization. J. Wood Sci..

[21] Li K., Wang S., Chen H., Yang X., Berglund L.A., Zhou Q. (2020). Self-Densification of Highly Mesoporous Wood Structure into a Strong and Transparent Film. Adv. Mater..

[22] Wang S., Chen H., Li K., Koskela S., Berglund L.A., Zhou Q. (2022). Strong, transparent, and thermochromic composite hydrogel from wood derived highly mesoporous cellulose network and PNIPAM. Compos. Part A Appl. Sci. Manuf..

[23] Yang Y., Dang B., Wang C., Chen Y., Chen K., Chen X., Li Y., Sun Q. (2023). Anisotropic Nature of Lightweight Wooden Metamaterials with Mechanical/Thermomechanical Multistability. Adv. Funct. Mater..

[24] Chen H. (2023). Preparation of Biomass-Based Aerogel Electrode Materials and Study on the Performance of Electrocatalytic Hydrogen and Oxygen Evolution. Master’s Thesis.

[25] Gao R. (2022). Preparation of Biomimetic Wood Aerogel and Its Application in the Field of Water Environment Purification. Ph.D. Thesis.

[26] He W., Qiang H., Liang S., Guo F., Wang R., Cao J., Guo Z., Pang Q., Wei B., Sun J. (2022). Hierarchically porous wood aerogel/polypyrrole(PPy) composite thick electrode for supercapacitor. Chem. Eng. J..

[27] Jin H., Nishiyama Y., Wada M., Kuga S. (2004). Nanofibrillar cellulose aerogels. Colloids Surf. A Physicochem. Eng. Asp..

[28] Sun C., Tan H., Zhang Y. (2023). Simulating the pyrolysis interactions among hemicellulose, cellulose and lignin in wood waste under real conditions to find the proper way to prepare bio-oil. Renew. Energy.

[29] Stubbs C., Baban N., Robertson D., Alzube L., Cook D., Geitmann A. (2018). Plant Biomechanics:From Structure to Function at Multiple Scales. Bending Stress in Plant Stems: Models and Assumptions.

[30] Fu Q., Ansari F., Zhou Q., Berglund L.A. (2018). Wood Nanotechnology for Strong, Mesoporous, and Hydrophobic Biocomposites for Selective Separation of Oil/Water Mixtures. ACS Nano.

[31] Shi J., Xing D., Lia J. (2012). FTIR Studies of the Changes in Wood Chemistry from Wood Forming Tissue under Inclined Treatment. Energy Procedia.

[32] Boukir A., Fellak S., Doumenq P. (2019). Structural characterization of *Argania spinosa* Moroccan wooden artifacts during natural degradation progress using infrared spectroscopy (ATR-FTIR) and X-Ray diffraction (XRD). Heliyon.

[33] Chen D., Zhou J., Zhang Q. (2014). Effects of Torrefaction on the Pyrolysis Behavior and Bio-Oil Properties of Rice Husk by Using TG-FTIR and Py-GC/MS. Energy Fuels.

[34] Collard F.-X., Blin J. (2014). A review on pyrolysis of biomass constituents: Mechanisms and composition of the products obtained from the conversion of cellulose, hemicelluloses and lignin. Renew. Sustain. Energy Rev..

[35] Soria-Verdugo A., Morgano M.T., Mätzing H., Goos E., Leibold H., Merz D., Riedel U., Stapf D. (2020). Comparison of wood pyrolysis kinetic data derived from thermogravimetric experiments by model-fitting and model-free methods. Energy Convers. Manag..

[36] Arseneau D.F. (1961). The differential thermal analysis of wood. Can. J. Chem..

[37] Yao W., Weng Y., Catchmark J.M. (2020). Improved cellulose X-ray diffraction analysis using Fourier series modeling. Cellulose.

[38] Abe K., Yamamoto H. (2005). Mechanical interaction between cellulose microfibril and matrix substance in wood cell wall determined by X-ray diffraction. J. Wood Sci..

[39] Li H., Zong Y., He J., Ding Q., Jiang Y., Li X., Han W. (2022). Wood-inspired high strength and lightweight aerogel based on carbon nanotube and nanocellulose fiber for heat collection. Carbohydr. Polym..

[40] Burgert I., Keplinger T. (2013). Plant micro- and nanomechanics: Experimental techniques for plant cell-wall analysis. J. Exp. Bot..

[41] Lu Y., Lu Y., Jin C., Gao R., Liu B., Huang Y., Yu Y., Ren J., Deng Y., Tao X. (2021). Natural Wood Structure Inspires Practical Lithium–Metal Batteries. ACS Energy Lett..

[42] Zhang X., Li L., Xu F. (2022). Chemical Characteristics of Wood Cell Wall with an Emphasis on Ultrastructure: A Mini-Review. Forests.

[43] Tsuguyuki S., Satoshi K., Yoshiharu N., Isogai A. (2007). Cellulose Nanofibers Prepared by TEMPO-Mediated Oxidation of Native Cellulose. Biomacromolecules.

[44] Tahiri C., Vignon M.R. (2000). TEMPO-oxidation of cellulose: Synthesis and characterisation of polyglucuronans. Cellulose.

[45] Xu F., Chen S., Zhang X. (2017). Combining Raman Imaging and Multivariate Analysis to Visualize Lignin, Cellulose, and Hemicellulose in the Plant Cell Wall. J. Vis. Exp..

[46] Gibson L.J. (1999). The Structure of Cellular Solids.

[47] Coste R., Soliman M., Bercu N.B., Potiron S., Lasri K., Aguié-Béghin V., Tetard L., Chabbert B., Molinari M. (2021). Unveiling the impact of embedding resins on the physicochemical traits of wood cell walls with subcellular functional probing. Compos. Sci. Technol..

