# Supplementary material for: Construction of Nanofibrillar Networked Wood Aerogels Derived from Typical Softwood and Hardwood: A Comparative Study on the In Situ Formation Mechanism of Nanofibrillar Networks
Source: Molecules. 2024 Feb 21;29(5):938. doi: 10.3390/molecules29050938 (PMC10935105; doi:10.3390/molecules29050938)
Supplement: Supplementary file 1 [file molecules-29-00938-s001.zip › molecules-2807056-supplementary.pdf]

Supplementary Materials

Construction of Nanofibrillar Networked Wood Aerogels Derived from Typical Softwood and Hardwood: A Comparative Study on the In situ Formation Mechanism of Nanofibrillar Networks

Wenjing Yan, Yan Qing, Zhihan Li, Lei Li, Sha Luo, Ying Wu, Deng Chen, Yiqiang Wu * and Cuihua Tian *

College of Materials Science and Technology, Central South University of Forestry and Technology, Changsha 410004, China

*Correspondence Authors

*Email: wuyiqiang@csuft.edu.cn (Yiqiang. Wu)；

*Email: tianch@csuft.edu.cn (Cuihua. Tian).

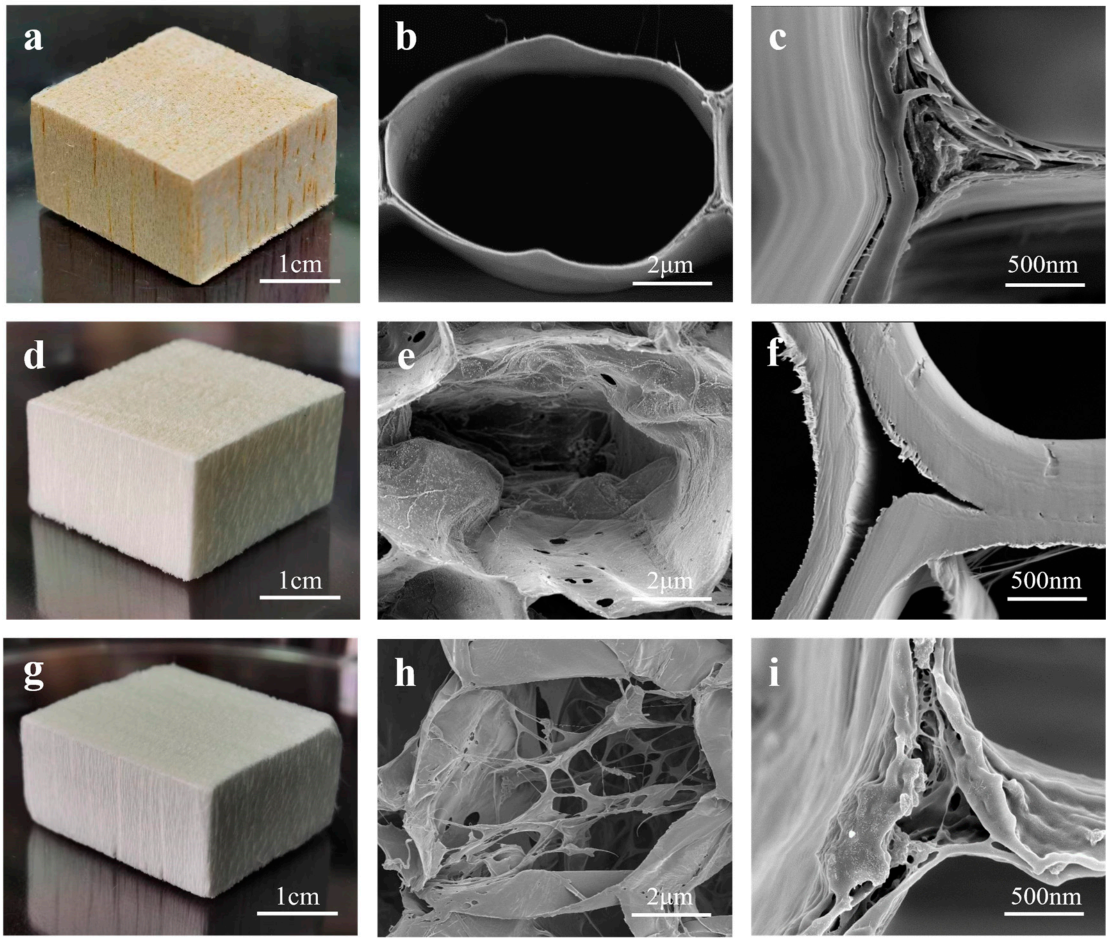

Figure S1. (a) Photograph of NW-3. **(b)** High magnification SEM image of NW-3 cell wall. **(c)** High magnification SEM image of NW-3 cell corner (CC). **(d)** Photograph of DW-3. **(e)** High magnification SEM image of DW-3 cell wall. **(f)** High magnification SEM image of DW-3 CC. **(g)** Photograph of WA-3. **(h)** High magnification SEM image of WA-3 cell wall. **(i)** High magnification SEM image of WA-3 CC.

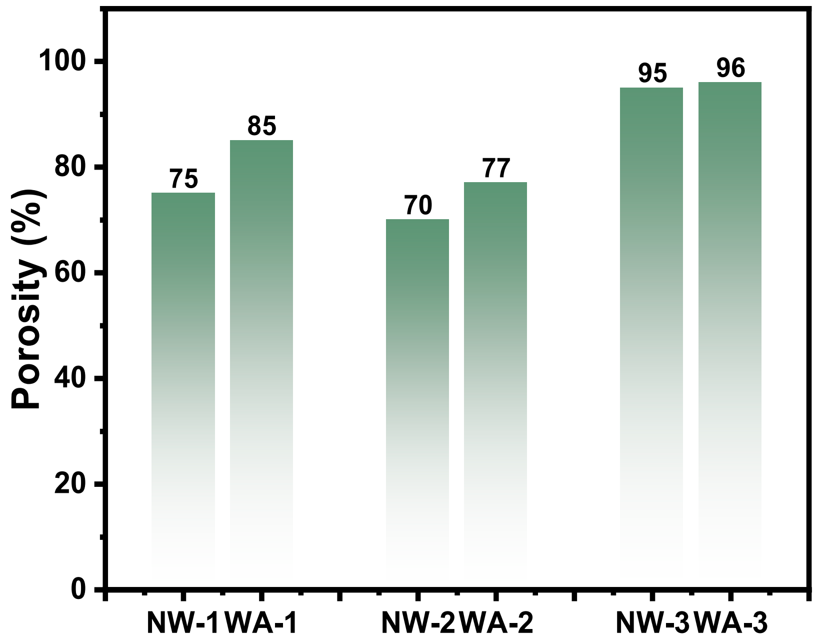

Figure S2. Porosity of NW-1, WA-1, NW-2, WA-2, NW-3, and WA-3.

The equation 1 was utilized to determine the porosity, with the solid density of wood assumed to be 1500 kg/m^3 [1].

$$\boldsymbol{porosity} = \mathbf{1} - \frac{\boldsymbol{density\ of\ sample}\ [\boldsymbol{kg}/\boldsymbol{m}^{\mathbf{3}}]}{\boldsymbol{solid\ density\ of\ sample}\ [\boldsymbol{kg}/\boldsymbol{m}^{\mathbf{3}}]} \quad (1)$$

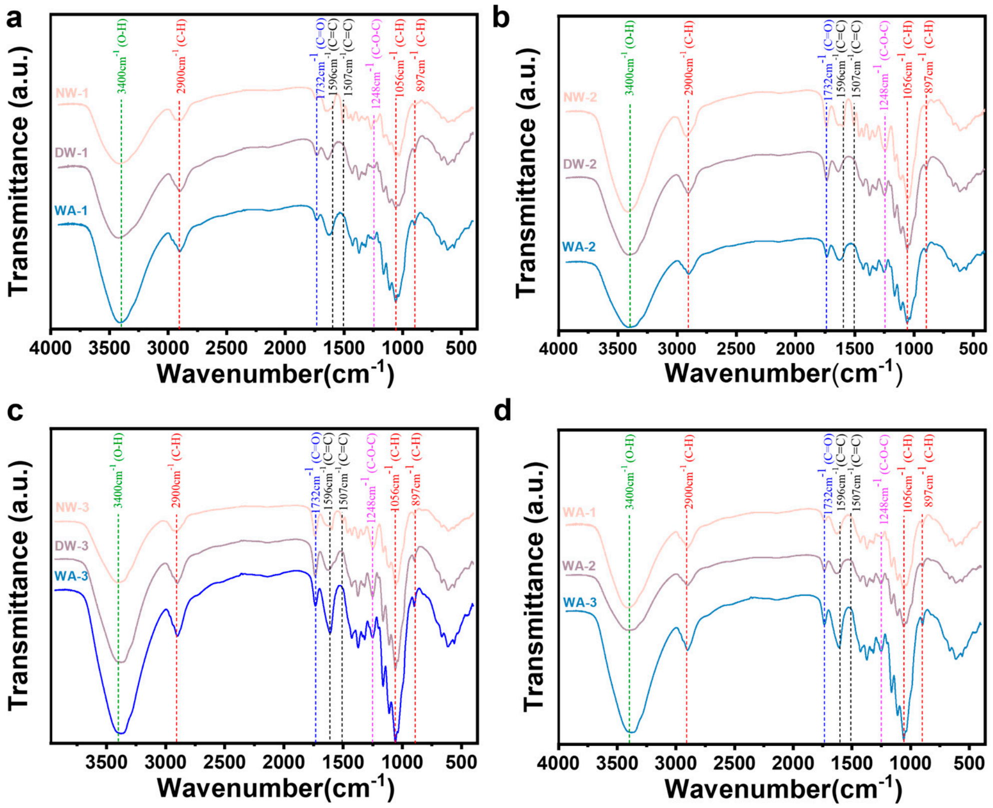

Figure S3. FT-IR spectra of **(a)** NW-1, DW-1 and WA-1. **(b)** NW-2, DW-2 and WA-2. **(c)** NW-3, DW-3 and WA-3. **(d)** WA-1, WA-2 and WA-3.

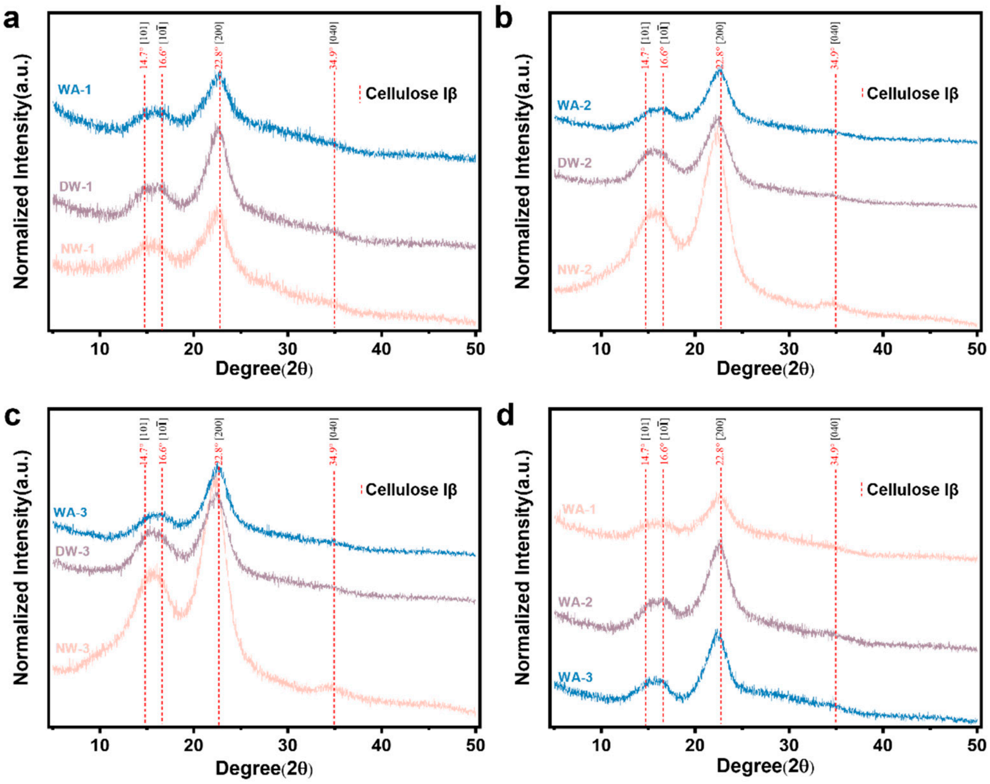

Figure S4. XRD spectra of **(a)** NW-1, DW-1 and WA-1. **(b)** NW-2, DW-2 and WA-2. **(c)** NW-3, DW-3 and WA-3. **(d)** WA-1, WA-2 and WA-3.

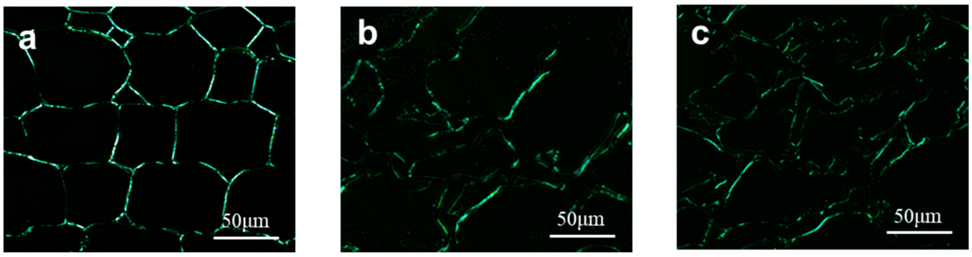

Figure S5. Polarizing microscopic image of **(a)** NW-3, **(b)** DW-3 and **(c)** WA-3.

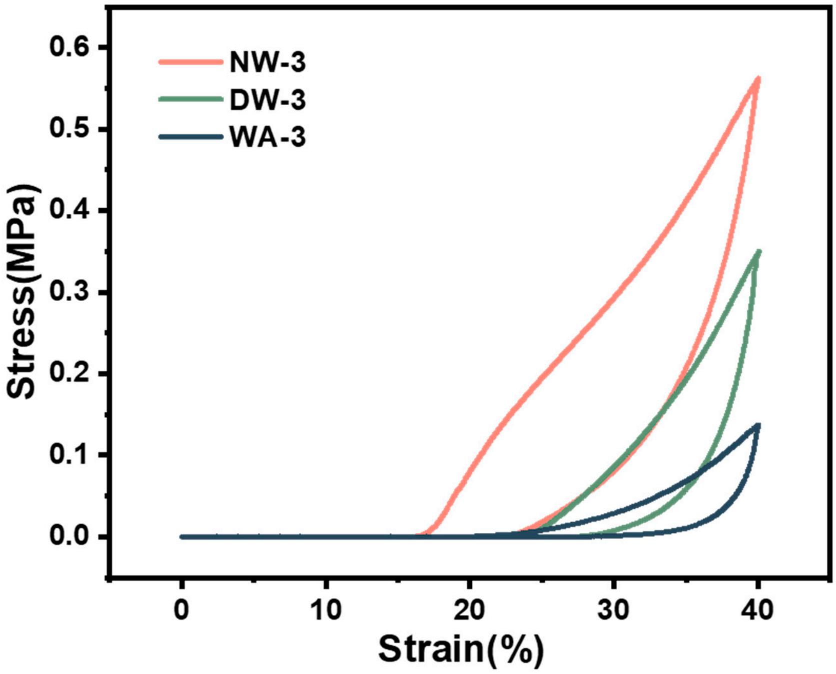

Figure S6. NW-3, DW-3 and WA-3 under cyclic compression at the maximum strain of 40%.

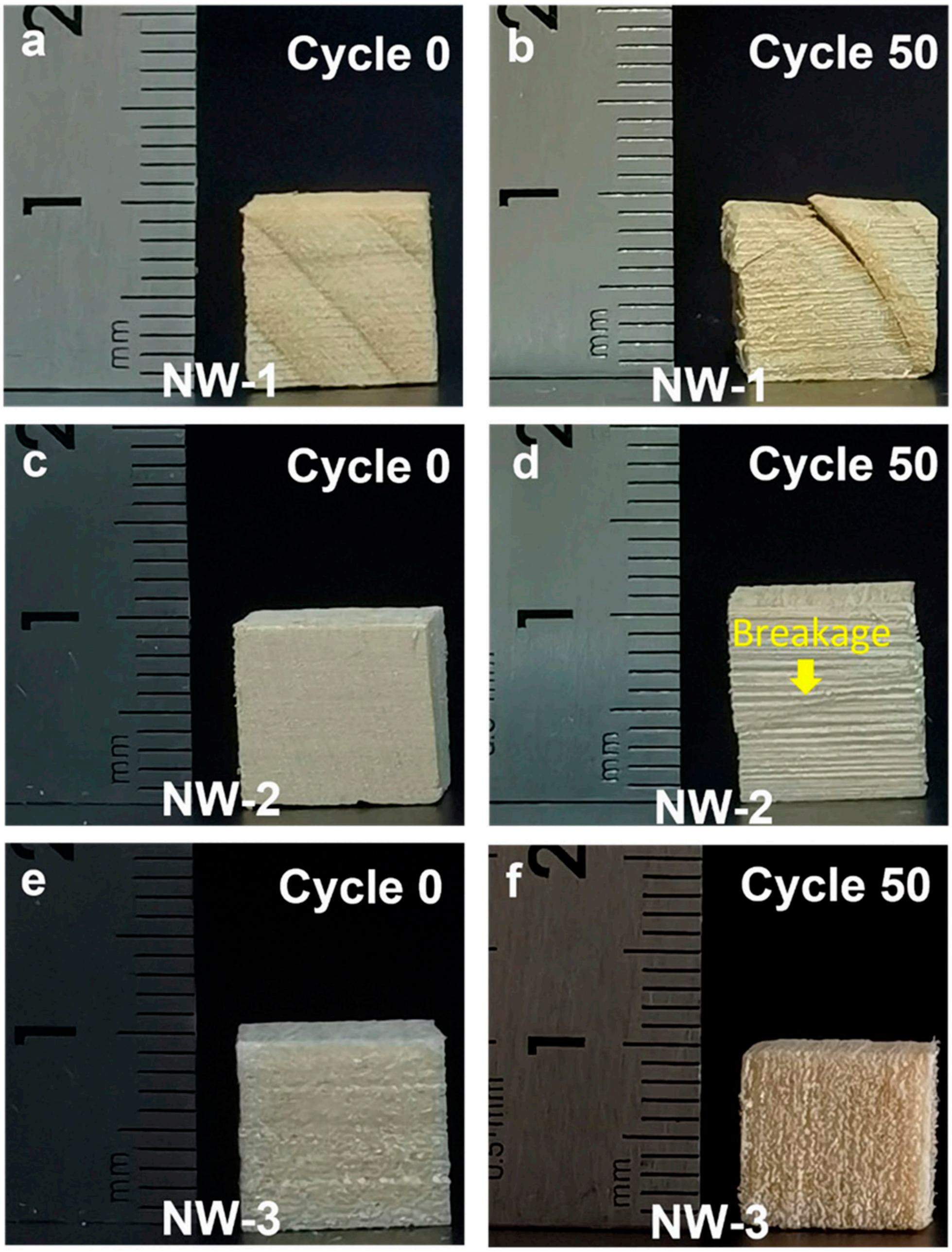

Figure S7. Mechanical compressibility of NWs. Photographs of the **(a)** NW-1 with 0 loading-unloading cycles. **(b)** NW-1 with 50 loading-unloading cycles. **(c)** NW-2 with 0 loading-unloading cycles. **(d)** NW-2 with 50 loading-unloading cycles. **(e)** NW-3 with 0 loading-unloading cycles. **(f)** NW-3 with 50 loading- unloading cycles.

Embedding slicing details:

The samples were sequentially immersed in ethanol solutions with concentrations of 25 wt%, 50 wt%, 75 wt%, and 100 wt%. After the displacement of the sample solution was completed, the samples were transferred to a mixture of Lr White Resin and ethanol, and then they were successively immersed in mixture solutions of Lr White Resin concentrations of 25 wt%, 50 wt%, 75 wt%, and 100 wt% until the displacement of the sample solution was completed. The samples immersed in 100 wt% Lr White Resin were transferred to a curing solution (Lr White Resin:curing agent = 10:1, V:V) and then placed in a suitable-sized embedding mold. The curing solution was poured into the mold to ensure that the samples were completely immersed in the embedding medium. To avoid the formation of bubbles, vacuum pumping could be used to remove air. If necessary, supporting materials such as wooden spatulas or plastic sheets could be used to assist in positioning and securing the samples. The samples were dried at 65 °C for 72 hours [2]. After further drying treatment of the embedded samples, they were sliced into semi-thin sections using a Leica rotary microtome [3].

Materials

Lr White Resin (contans 5ppm hydroquinone) and curing agent was provided by Head (Beijing) Bio-Technology Co., Ltd. (Beijing, China). Ethanol (99.7%, CH_3CH_2OH) was provided by HengXing Chemical Reagent Co., Ltd. (Tianjin, China), and all chemicals were used of analytical grade.

References

1. Gibson, L.J. The Structure of Cellular Solids; Cambridge University Press: Cambridge, UK, 1999; pp. 15–51. https://doi.org/10.1017/CBO9781139878326.

2. Coste, R.; Soliman, M.; Bercu, N.B.; Potiron, S.; Lasri, K.; Aguié-Béghin, V.; Tetard, L.; Chabbert, B.; Molinari, M. Unveiling the impact of embedding resins on the physicochemical traits of wood cell walls with subcellular functional probing. *Compos. Sci. Technol.*, **2021**, *201*, 108485. http://doi.org/10.1016/j.compscitech.2020.108485.

3. Garemark, J.; Perea-Buceta, J.E.; Felhofer, M.; Chen, B.; Cortes Ruiz, M.F.; Sapouna, I.; Gierlinger, N.; Kilpelainen, I.A.; Berglund, L.A.; Li, Y. Strong, Shape-Memory Lignocellulosic Aerogel via Wood Cell Wall Nanoscale Reassembly. *ACS Nano* **2023**, *17*, 4775–4789. https://doi.org/10.1021/acsnano.2c11220.
